# A low-cost didactic module for testing advanced control algorithms

**DOI:** 10.1016/j.ohx.2020.e00148

**Published:** 2020-10-09

**Authors:** Omar Gustavo Celso Pinares-Mamani, Juan C. Cutipa-Luque

**Affiliations:** Universidad Nacional de San Agustín de Arequipa, Arequipa, Peru

**Keywords:** Cart inverted pendulum, Didactic module, 3D printer, Control systems.

## Abstract

Commercial modules for learning advanced control systems are not quite common and some are very expensive due to their sensors, electronic and license. There is an open area to develop and to build didactic modules to improve the learning process using experimentation in real physical systems. Particularly, a cart inverted pendulum is a classical physical system very commonly used in recent decades. We propose a cart inverted pendulum named MoDiCA-X as a low-cost didactic module with open source hardware and software. It is an electromechanical system feasible to build and easy to be modified. The mechanical parts of the module are 3D printed solids and can also be easily replicated. In terms of programming, the control applied to the system can be modified, since it uses C/C++ programming languages that are widely used in the academic community. The module is equipped with two very commercial sensors, are easy to install and to remove; both acquire the pendulum attitude and the car position. The actuators are four electric DC motors coupled to the car wheels to provide suitable velocity and torque to each axle independently. We validate the performance of the module by applying a multivariable linear quadratic regulator algorithm (LQR).

**Specifications table:**

**Table:** 

Hardware name	MoDiCA-X
Subject area	Electrical engineering
	Control system engineering
Hardware type	Learning didactic modules
	Nonlinear dynamic and control
Open source license	GNU GPL v3
Cost of hardware	102.95 $ US
Source file repository	https://doi.org/10.17605/OSF.IO/JS9B2

## 1 Hardware in context

There is a continuous challenge for instructors and students to experimentally perceive how advanced control algorithms work. In the early 2010s, for example, two papers highlighted the challenge and the possible solution supported in commercial Lego Mindstorms NXT Kits [1], [2]. This alternative is still too expensive for many academic institutions to afford and is not an optimal investment due to the limited features imposed by copyright restrictions. Another option is to implement fabrication laboratories (Fab Lab) with 3D printers, laser cutting machines, and printed circuit board machines to facilitate fabrication, so that they can build their own physical systems and robots [3], [4].

Inverted pendulum is currently a system used to validate novel approaches in control engineering. In [5], for example, the authors show an experimental pendulum module to validate their event-triggered fuzzy control approach, which can be applied to nonlinear systems. In [6], [7], the authors present an inverted pendulum with modeling and advanced control approaches validated experimentally.

There are several types of inverted pendulums; one of them is the balance robot, used as a learning tool for students. Conventional PID control approach in ADROIT V1 educational robot kits is presented in [8]. For academic purposes, an inverted pendulum is presented in [9], which is a low-cost two-wheels self-balancing robot and is implemented on Arduino platform. Advanced control approaches are nowadays used in many electronic equipment, and lead the trend topics in research areas seeking to improve the performance in complex systems. The prototype widely used for testing these advanced control approaches is also the inverted pendulum. In [10], the authors apply a multivariate control approach, based on a modified energy Lyapunov functions, which is implemented and analyzed in real time. Another control approach is the robust transition control of a self-balancing vehicle [11], which is a self-balancing personal vehicle, named Segway. Didactic modules to improve the learning and to validate novel approaches in control engineering are needed and can be fabricated straightforwardly using the tools and equipment of fabrication laboratories available in academic institutions. The goal of this work is to supply the necessity of academics in testing experimentally advanced control algorithm for real physical system, under-actuated, non minimum phase class, linear and nonlinear. There are several advantages of using MoDiCA-X respect to other modules presented in [9], [10], [11], [12]; because it is a low-cost, completely open source, portable, flexible to implement algorithm according to user requirement, easy to replicate and does not tie with commercial licenses. The MoDiCA-X is a didactic module for advanced control with open source features as described next.

## 2 Hardware description

**Fig. 1:**
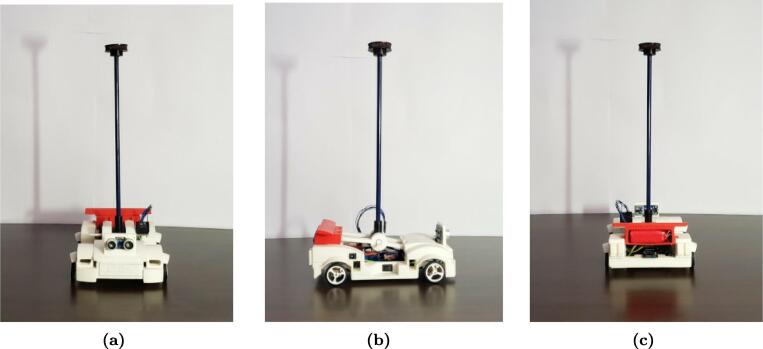
MoDiCA-X didactic module for advanced control: (a) frontal view, (b) lateral view and (c) rearview.

This work presents a didactic module or prototype for teaching advanced control theory through simple experiments consisting of a pendulum on a cart with dimensions of 0.214×0.154×0.365 m, developed with mechanical and electronic components. The motivation to develop this hardware is making the control system discipline more attractive and for students to intuitively understand stability and performance concepts through recorded data. Fig. 1 presents the final version of the prototype in frontal view (a), lateral view (b) and rearview (c).

The functionalities of this module can be summarized as follows:

• A low-cost and rapid prototype using minimal material and commonly Fab Lab equipment.
• Flexible to test multiple algorithms of control, since the pendulum car is a main example used in the literature, from basic to advanced control systems.
• Modularity enables integrating the electronic hardware components, such as memory card, to record data, serial peripheral to connect with a computer to set the parameters or to upload new algorithms.

The prototype comprises both mechanical and electronic components its hardware. The mechanical parts can be manufactured using common materials, such as aluminum, wood and 3D-printed solids. The electronic parts can be implemented using low-cost electronic devices contained in a printed circuit board (PCB). The prototype total weight is 0.65 kg and can carry a maximum mass of 0.60 kg on top without compromising its structure.

**Fig. 2:**
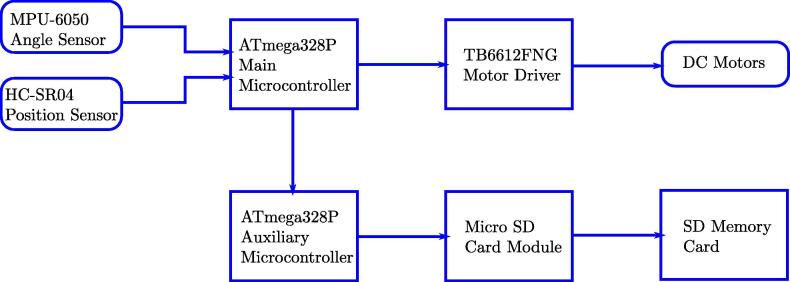
Block diagram of the MoDiCA-X.

Fig. 2 presents the integration of electrical parts of the system in block diagram. The core is the main microcontroller ATmega328P; the car position sensor is an ultrasonic device HC-SR04; the pendulum angle sensor is a microelectromechanical system (MEMS) MPU-6050; the data-logger is composed of a secure digital (SD) memory card of 2 GB (gigabytes) and another auxiliary microcontroller ATmega328P. The MPU-6050, one of the low-cost inertial measurement unit, is commonly used in small aerial unmanned vehicles and its specifications are sufficient for the pendulum system. The HC-SR04 is another low-cost sensor for position that guarantees the requirement for the car dynamic in surge direction. The module presents four independent 6 V DC motors attached to the car wheels, a driver TB6612FNG commanded with the main microcontroller to feed the motors with the necessary energy. The main microcontroller reads the sensor data, executes the advanced control algorithms, and produces actuator signal for the motor power driver. In parallel, the auxiliary microcontroller communicates with the main microcontroller for sharing sensors data and other variables to be saved in a micro SD card. This distributed architecture is necessary to reduce interrupt latency and minimal thread switching latency.

## 3 Design files

### 3.1 Hardware files

**Table:** 

Design filename	File type	Open source license	Location of the file
Chassis.STL	STL Solidworks	GPL 3:0	https://osf.io/ngb3u/
Right_Bearing_Brackets.STL	STL & Solidworks	GPL 3.0	https://osf.io/8nbwz/
Left_Bearing_Brackets.STL	STL & Solidworks	GPL 3.0	https://osf.io/3jqna/
Rear_Spoiler.STL	STL & Solidworks	GPL 3.0	https://osf.io/x7kr4/
Pendulum.STL	STL & Solidworks	GPL 3.0	https://osf.io/8ajt7/
T-tube.STL	STL & Solidworks	GPL 3.0	https://osf.io/qre98/
Axle.STL	STL & Solidworks	GPL 3.0	https://osf.io/f4vp2/

• Chassis.STL: The main structure of the 3D printed module containing the PCB, wheels, and motors.
• Right_Bearing_Brackets.STL and Left_Bearing_Brackets.STL: 3D printed parts attached to the spoiler and chassis, contains the bearings and the pendulum axle.
• Rear_Spoiler.STL: 3D printed part attached to the chassis that contains the battery.
• Pendulum.STL: 3D printed part of the aluminum rod.
• T-tube.STL: 3D printed piece that joins the aluminum rod and the pendulum axle.
• Axle.STL: 3D printed part that supports the aluminum rod; it also contains the MPU-6050 sensor.

### 3.2 Software files

**Table:** 

Design filename	File type	Open source license	Location of the file
Main_code.ino	Arduino Sketch	GPL 3.0	https://osf.io/3w679/
Auxiliary_code.ino	Arduino Sketch	GPL 3.0	https://osf.io/ctdj4/

• Main_code.ino: Main program of the MoDiCA-X which runs the control algorithm system, reading sensor MPU-6050 and HC-SR04 values and delivers signals to the motor power driver.
• Auxiliary_code.ino: Code of the auxiliary microcontroller to record sensor data in a micro SD card.

## 4 Bill of materials

• MoDiCA-X: https://osf.io/2my7w/

**Table:** 

Designator	Component	Number	Cost per unit currency	Total cost	Source of materials	Material type
Microcontroller	ATmega328P on Arduino Nano	2	$ 9.72	$ 19.44	https://amzn.to/2RRyQOc	Others
Battery	7.4 V/2S 1000 mAh 20 C Lipo battery JST Plug	1	$ 11.00	$ 11.00	https://amzn.to/2ucDflB	Others
DC Motor	Motor N20 6 V 1000 RPM	4	$ 8.99	$ 35.96	https://bit.ly/2NmLEZD	Others
Tires	34 mm wheel for DC 12 V N20 micro motor	2	$ 7.90	$ 15.80	https://bit.ly/3b1M9my	Others
Position sensor	HC-SR04	1	$ 5.95	$ 5.95	https://bit.ly/31i6Whe	Others
Angle sensor	MPU-6050	1	$ 4.80	$ 4.80	https://amzn.to/392ePtK	Others
Motor driver	TB6612FNG	1	$ 5.45	$ 5.45	https://bit.ly/36Q0ZJD	Others
Voltage Regulator	Regulator 7805	1	$ 0.98	$ 0.98	https://bit.ly/2ugBPq6	Others
Male connector pins	40 Pin 2.54 mm male pin header	2	$ 0.10	$ 0.20	https://bit.ly/2tjdKyu	Others
Female connector pins	40 Pin, 2.54 mm female pin header	2	$ 0.10	$ 0.20	https://bit.ly/37Tmz10	Others
Voltage regulator input capacitor	0.33 uF, 100 V electrolytic capacitor	1	$ 0.34	$ 0.34	https://bit.ly/2uYQ0jA	Others
Voltage regulator output capacitor	0.1 uF 50 V electrolytic capacitor	1	$ 0.36	$ 0.36	https://bit.ly/36OJcm8	Others
Terminal blocks	2-way terminal block	3	$ 0.10	$ 0.30	https://bit.ly/2GPyNvI	Others
Switches	Mini rocker switch 2 PIN ON–OFF	2	$ 0.10	$ 0.20	https://bit.ly/2SfXzux	Others
Resistor	Carbon Film Resistor 1 K Ohm 1/4 W	1	$ 0.01	$ 0.01	https://bit.ly/36TxqGS	Others
Jumper	Female to female jumper wire	40	$ 0.01	$ 0.40	https://bit.ly/2Ujm4JS	Others
Micro SD memory	2 GB Micro SD memory	1	$ 0.80	$ 0.80	https://bit.ly/2UGKZX4	Others
Micro SD Module	Micro SD Module	1	$ 0.70	$0.70	https://bit.ly/3hxia9x	Others
DIP switch 4	DIP switch 4	1	$0.06	$ 0.06	https://bit.ly/2B2xfz4	Others

## 5 Building instructions

### 5.1 Electronic circuit assembly

**Fig. 3:**
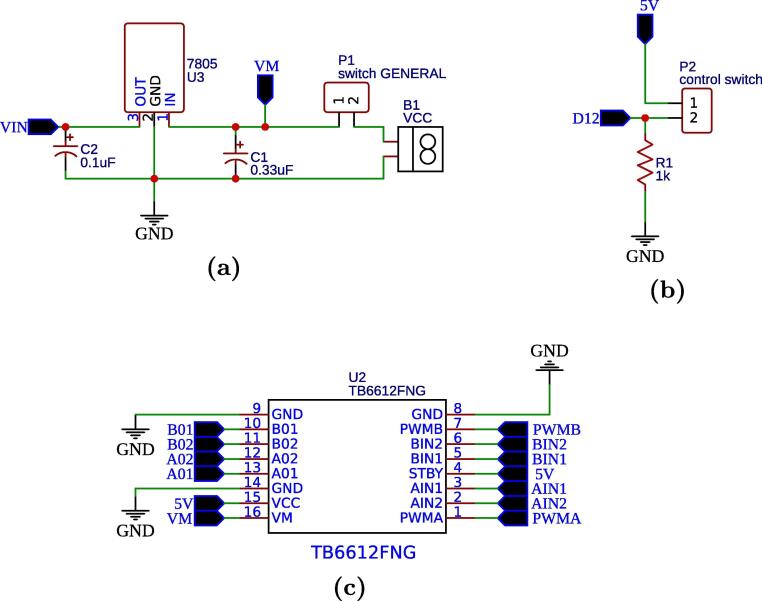
Power supply and driver circuits: (a) regulator, (b) pulldown and (c) driver TB6612FNG.

**Fig. 4:**
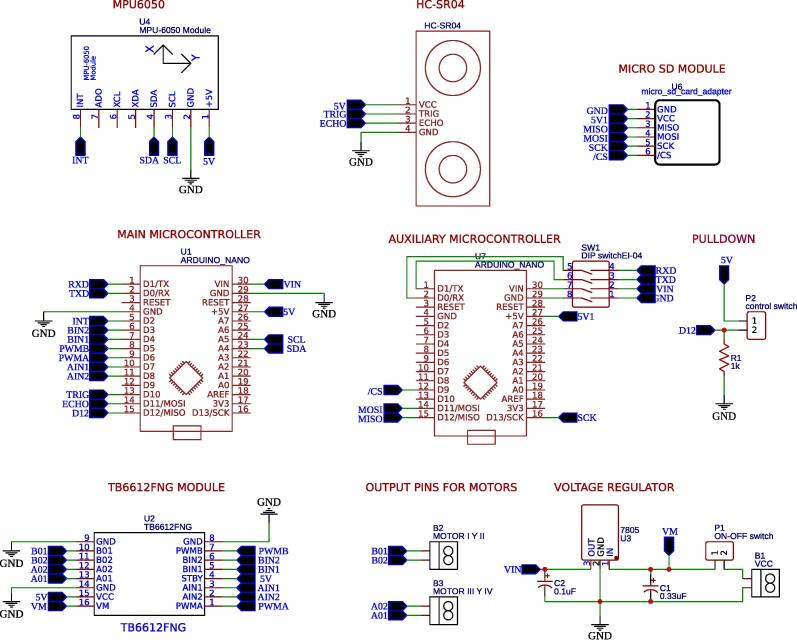
Schematic circuit of MoDiCA-X.

The input circuit is composed of a voltage regulator 7805 and devices according to the schematic shown in Fig. 3a. This circuit regulates the input voltage from the battery to 5 V, which will feed the ATmega328P microcontrollers through the VIN pin (Fig. 4). The ATmega328P has a 5 V pin that is a power supply for electronic devices such as the MPU-6050, HC-SR04, micro SD module, TB6612FNG and also for the pulldown circuit (Fig. 3b). The TB6612FNG module has a VM pin (Fig. 3c) used to power the motors, which is connected to a node at the regulator input, since the motors work with a voltage higher than 5 V.

**Fig. 5:**
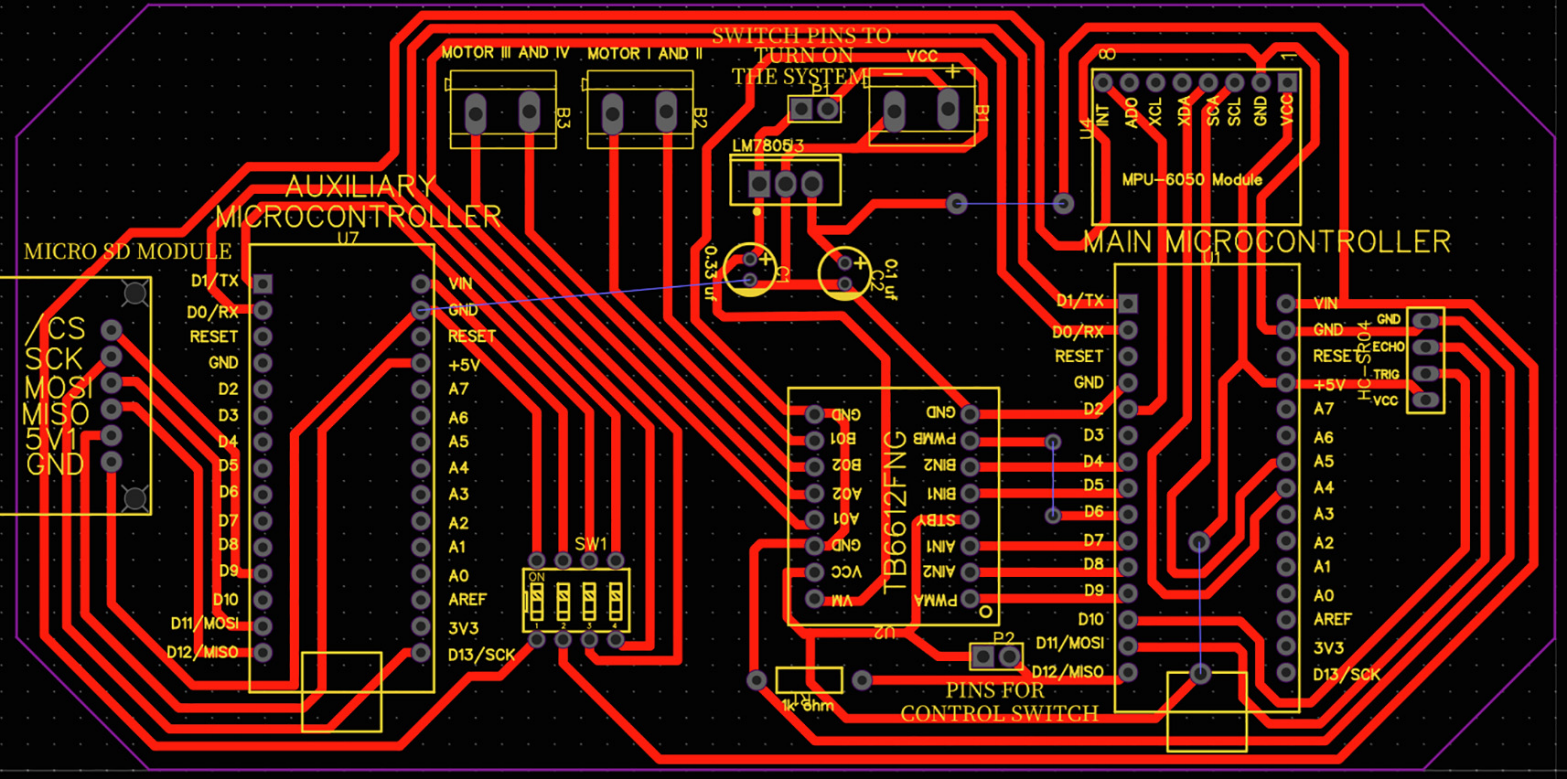
Final printed circuit board (PCB) design of MoDiCA-X.

**Fig. 6:**
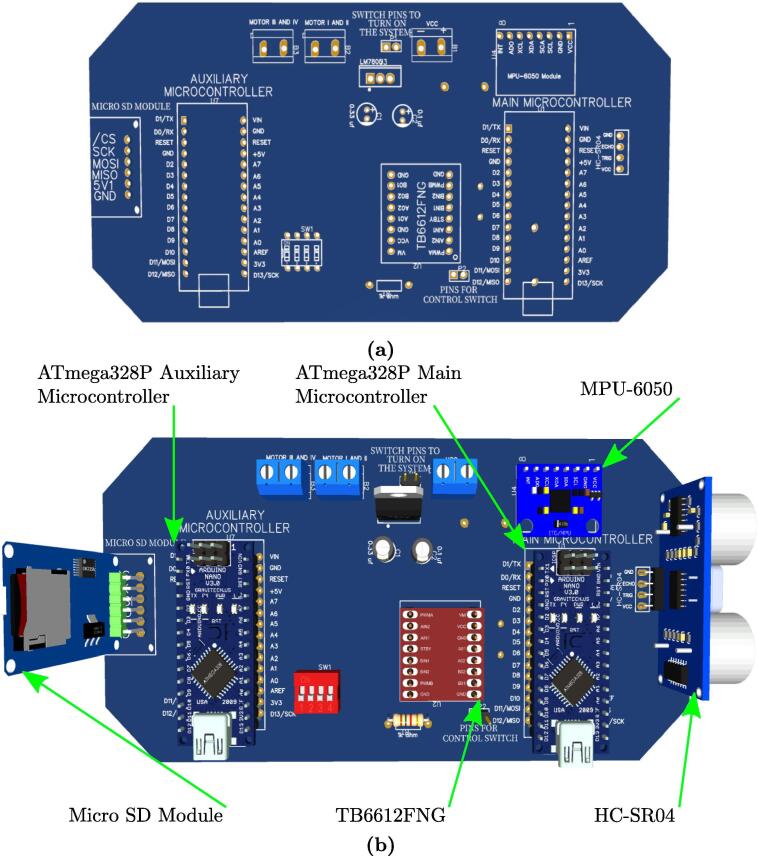
3D view of the PCB: (a) PCB solder mask (b) 3D view.

For the electronic design and schematic, the EasyEDA software is used; it is available for free at https://easyeda.com/es. This tool has shown good features and facilities recommended for beginner and intermediate users. Fig. 4 shows the schematic of all the components used in MoDiCA-X, naming their pins according to proper interconnections. The PCB is easily obtained using the same software. Fig. 5 shows the final results, while Fig. 6 shows a 3D view of the printed circuit with specific location of the electronic devices and components.

• MoDiCA-X Schematic: https://osf.io/7v2q5/
• MoDiCA-X PCB: https://osf.io/9mb6f/

**Fig. 7:**
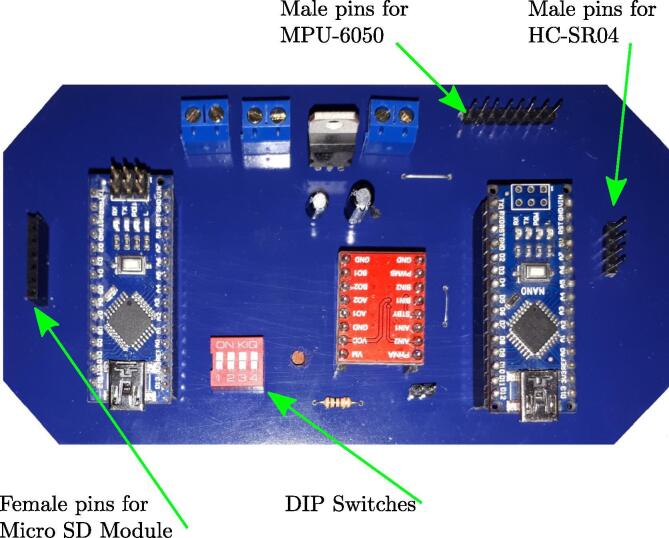
Real picture of the final printed circuit board.

**Fig. 8:**
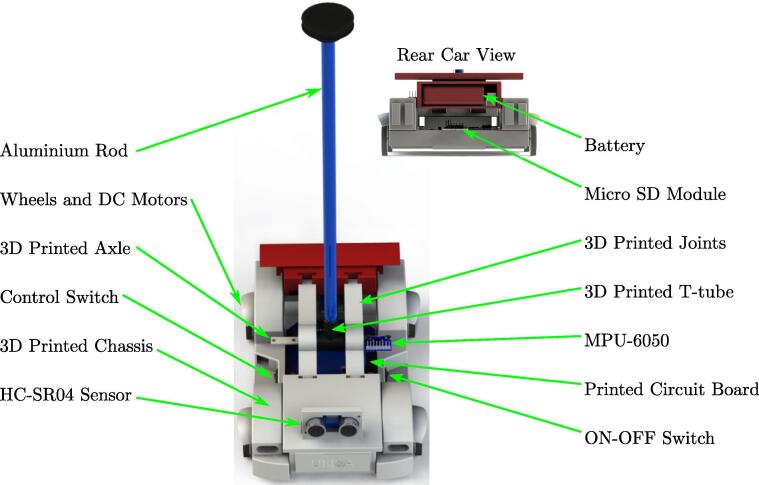
Full components of the didactic module MoDiCA-X.

The MPU-6050, the HC-SR04 and SD card module are located externally to the PCB and connected by connectors. The MPU-6050 is attached to the pendulum axle to measure the angle; the HC-SR04 is attached to the front bumper and the SD card module is on the external car chassis for easy manipulation, as shown in Fig. 8. The printed circuit board in Fig. 7 shows two male and one female pin arrays to connect these devices.

• PCB solder mask: https://osf.io/2feh5/
• 3D view: https://osf.io/kecpw/
• Printed circuit board: https://osf.io/9kgn7/

### 5.2 Mechanical and auxiliary component assembly

Fig. 8 shows the full assembly of the didactic module describing its parts. The PCB is on the car chassis and the pendulum structure is close to the gravity center supported by two joints and using two bearings in order to reduce friction. The pendulum is composed of aluminum and the battery is attached to the car rear. It is very compact and easy to transport from desk to operate in any laboratory, classroom or outdoors.

**Fig. 9:**
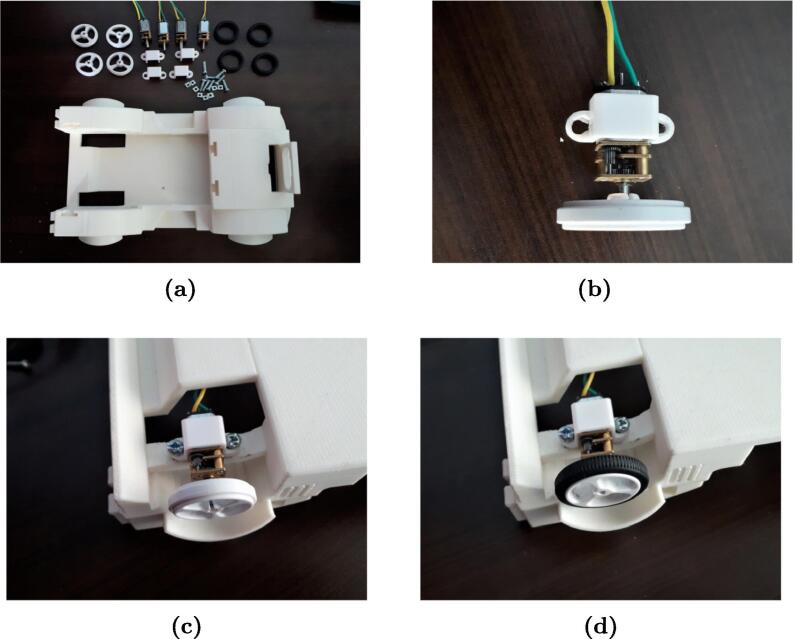
Instruction for wheel assembly: (a) print 3D solids and clamps, (b) insert the DC motor into the wheel axle, (c) attach the DC motor to the car chassis and (d) cover the wheel with a tire.

**Fig. 10:**
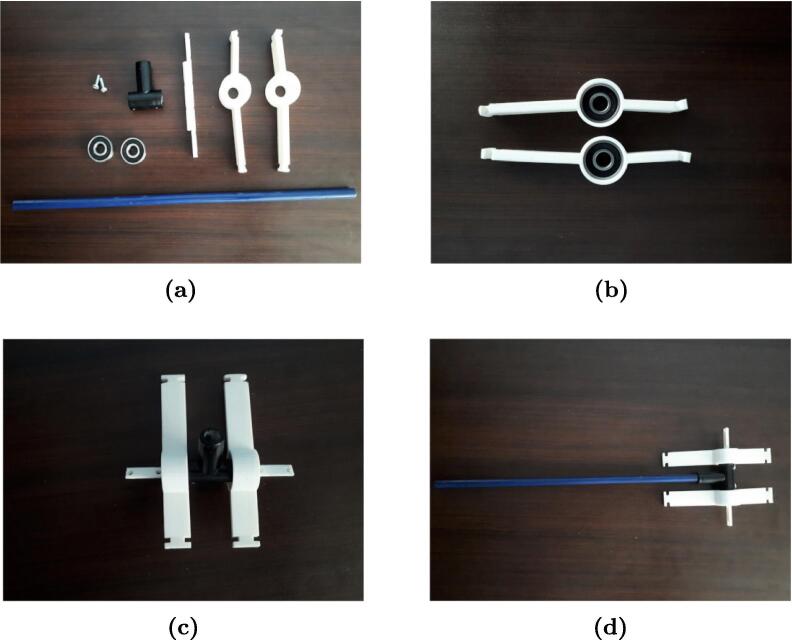
Instruction for pendulum assembly: (a) print the 3D solids, (b) insert the bearings into the joint supports, (c) insert the axle into the T-tube support, (d) insert the pendulum aluminum rod into the hole of the T-tube.

The full structure is composed of 3D printed solids with PLA type filament, widely used by 3D printers due to their low-cost. All the parts are printed separately as shown in Fig. 9, Fig. 10, and finally assembled as described above.

#### 5.2.1 Wheel assembly instruction

MoDiCA-X uses 4 wheels covered by rubber tires, each with its respective 1000 rpm N20 DC motor, commonly used in mobile robot competition due to its high torque [13]. The speed of the motor will depend on the control signal sent by the ATmega328P. Fig. 9a shows the procedure for fitting the wheel to the axles. Fig. 9b shows a motor assembly with a wheel and how to fix it using clamps and screws. Fig. 9c shows how to insert the motor support in the car chassis including how to properly insert the connectors. Fig. 9d shows a tire fully assembled to the module.

#### 5.2.2 Pendulum assembly instruction

Two bearings, model 608-2RS-C3, are used for the pendulum mobility and to reduce friction due to the axle rotation of the T-tube support. Fig. 10 shows the procedure for assembling the pendulum. Fig. 10a shows the necessary parts to assemble the pendulum and the MPU-6050 sensor. Fig. 10b shows how to insert the bearings in the hole joints. Fig. 10c shows how to insert the pendulum axle in the supports and the T-tube piece.

#### 5.2.3 Main structure assembly instruction

**Fig. 11:**
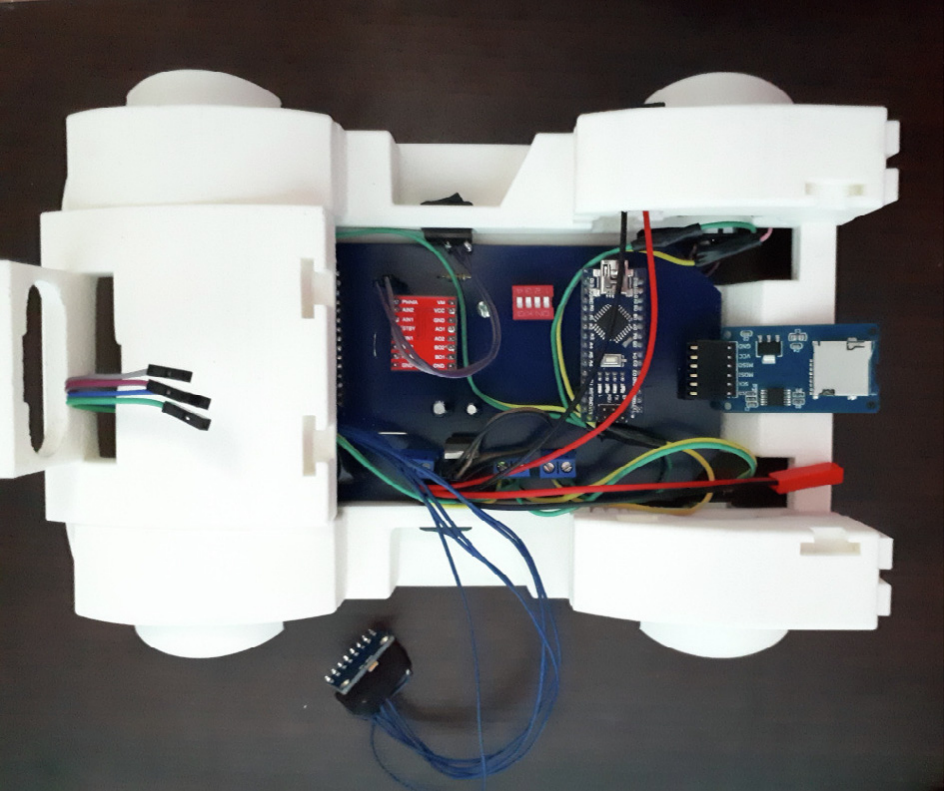
Connection instruction: locate and connect the MPU-6050 sensor, the micro SD module, the DC motors, the switches and the power jack in the PCB.

**Fig. 12:**
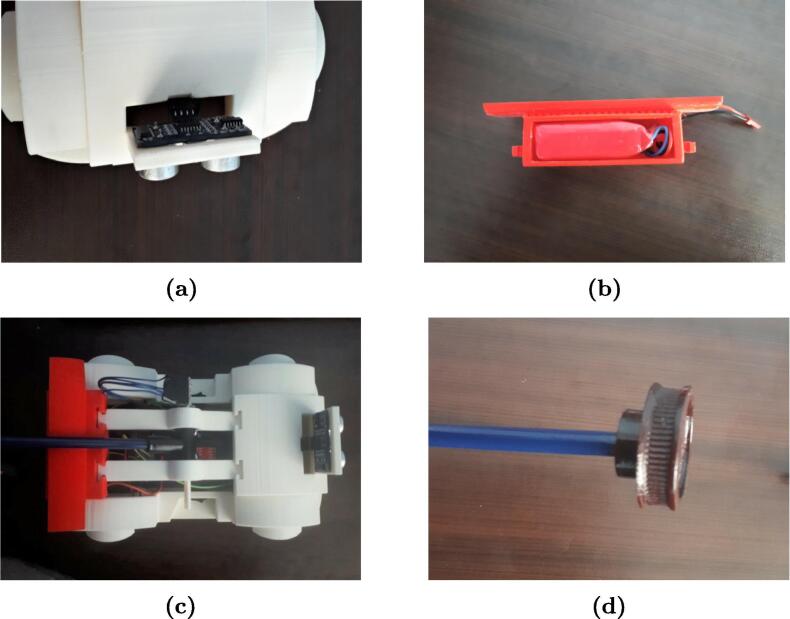
Instruction for MoDiCA-X assembly: (a) put the HC-SR04 sensor into the bumper and insert the connectors into the proper pins, (b) insert the battery below spoiler, (c) mount the pendulum joint supports between the spoiler and the middle of the car, and (d) put the pendulum rod into the T-tube.

Fig. 11, Fig. 12 show the assembly instructions for the main structure, which includes sensors, battery, switches, pendulum top base, micro SD module and the printed circuit board. Fig. 12 details how to mount the micro SD module, MPU-6050 sensor and the switches inside the car, as well as the connections between the devices as shown in Fig. 5, Fig. 6. The HC-SR04 position sensor is installed in the car bumper, the battery is inserted below the spoiler, and the MPU-6050, in the main pendulum axle.

## 6 Operation instructions

### 6.1 Calibration of MPU-6050 sensor

The module uses the MPU-6050 sensor that measures the position of the pendulum; it is very important to define the sensor set-point where the pendulum is perpendicular to the car base. Therefore, before starting the module operation, users are recommended to implement the following calibration procedure for the MPU-6050 sensor:

1. Download the MPU-6050 sensor calibration code: https://osf.io/v34js/
2. Load the code into the main microcontroller (Fig. 6).

**Fig. 13:**
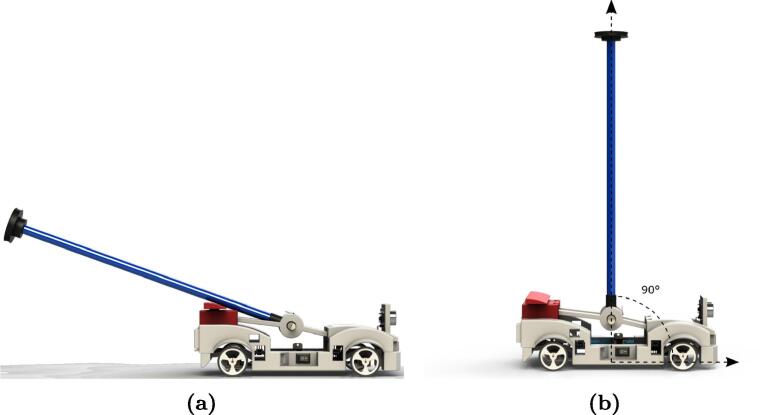
Calibration of MPU-6050 sensor: (a) pendulum at rest and (b) pendulum in the vertical position.
3. Place the pendulum in the rest position as seen in Fig. 13b, for at least 15 s.
4. Open the monitor in series and place the pendulum vertically or perpendicularly to the carriage base as shown in Fig. 13b.
5. Then, write down on a sheet the value measured by the MPU6050 sensor when it is upright.

It is very important to write down the measured value, because it will later be used in the main program of MoDiCA-X.

### 6.2 Calibration of HC-SR04 sensor

The HC-SR04 sensor performs an important function in the didactic module since it measures the car position and it is mandatory to define a set point for the sensor. Unlike the other sensor, it has a variable set point between 10 cm and 30 cm, which means, users can choose any value within that range after following the calibration procedure below:

1. Download the HC-SR04 sensor calibration code: https://osf.io/5snd6/
2. Load the code into the main microcontroller (Fig. 6).
3. Place the car in front of a vertical flat surface regardless of whether the pendulum is resting or in the vertical position as shown in Fig. 14.

**Fig. 14:**
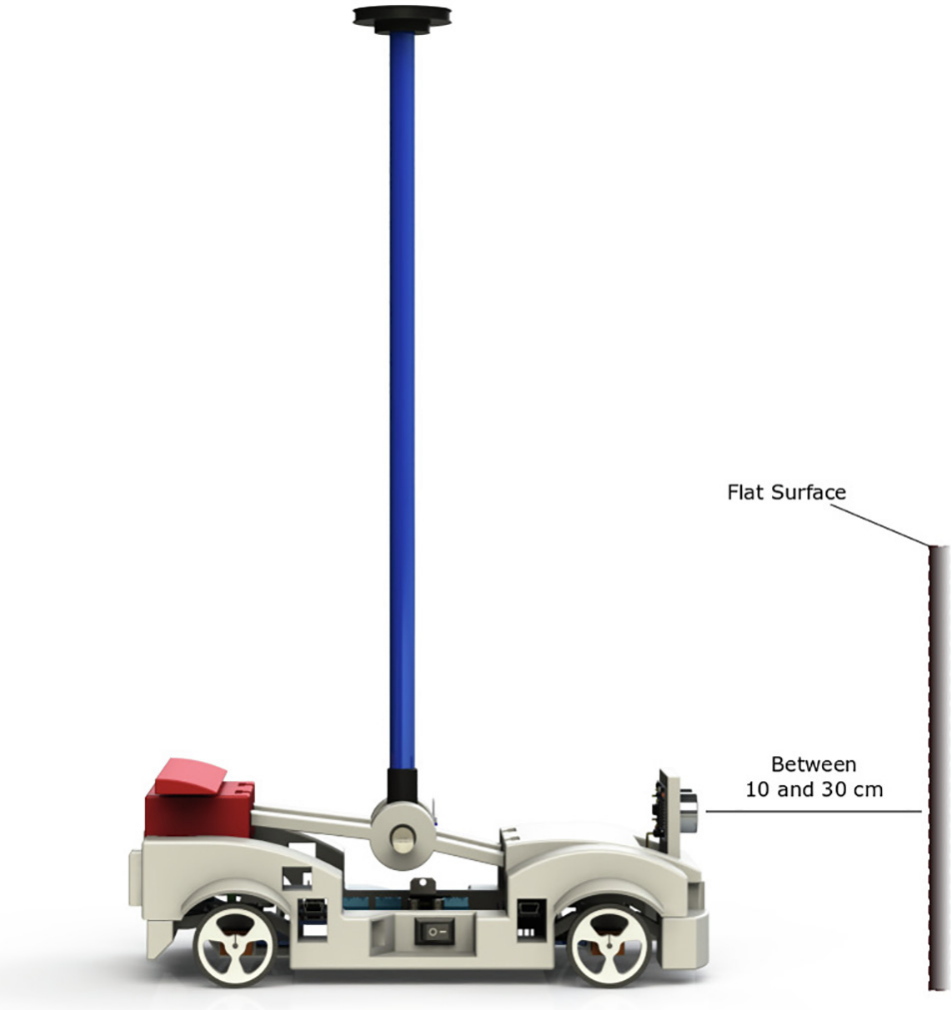
Calibration of position HC-SR04 sensor.
4. Open the serial monitor, then move the car towards the vertical surface to sense distance.
5. Make a note of the position value you choose taking into account the limits between 10 cm and 30 cm.

### 6.3 Instruction for using MoDiCA-X

When the module is fully implemented, the user may follow the procedure below:

1. Download the main code. Section 3.2.
2. Modify the set point values obtained in the calibration procedure of two sensors, through variables VALHCSR04 and VALMPU6050 located in the first section of the main program.
3. Set all the switches (DIP Switch 4) to OFF.
4. Load the main code and compile the modified main program (Fig. 6).
5. Insert the SD memory into the micro SD module.
6. Download the auxiliary code Section 3.2.
7. Load the auxiliary code for the auxiliary microcontroller (Fig. 6).
8. Set all switches (DIP Switch 4) to ON.

### 6.4 Operation procedure

1. Put the module on a surface, such as a table, and in front of a vertical flat surface, such as a wall.
2. Press the ON–OFF switch to turn on the module.
3. Put the pendulum in a rest position, around 15 degrees relative to the vertical axis.
4. Press the control switch to activate the advanced control algorithm.

## 7 Validation and characterization

**Fig. 15:**
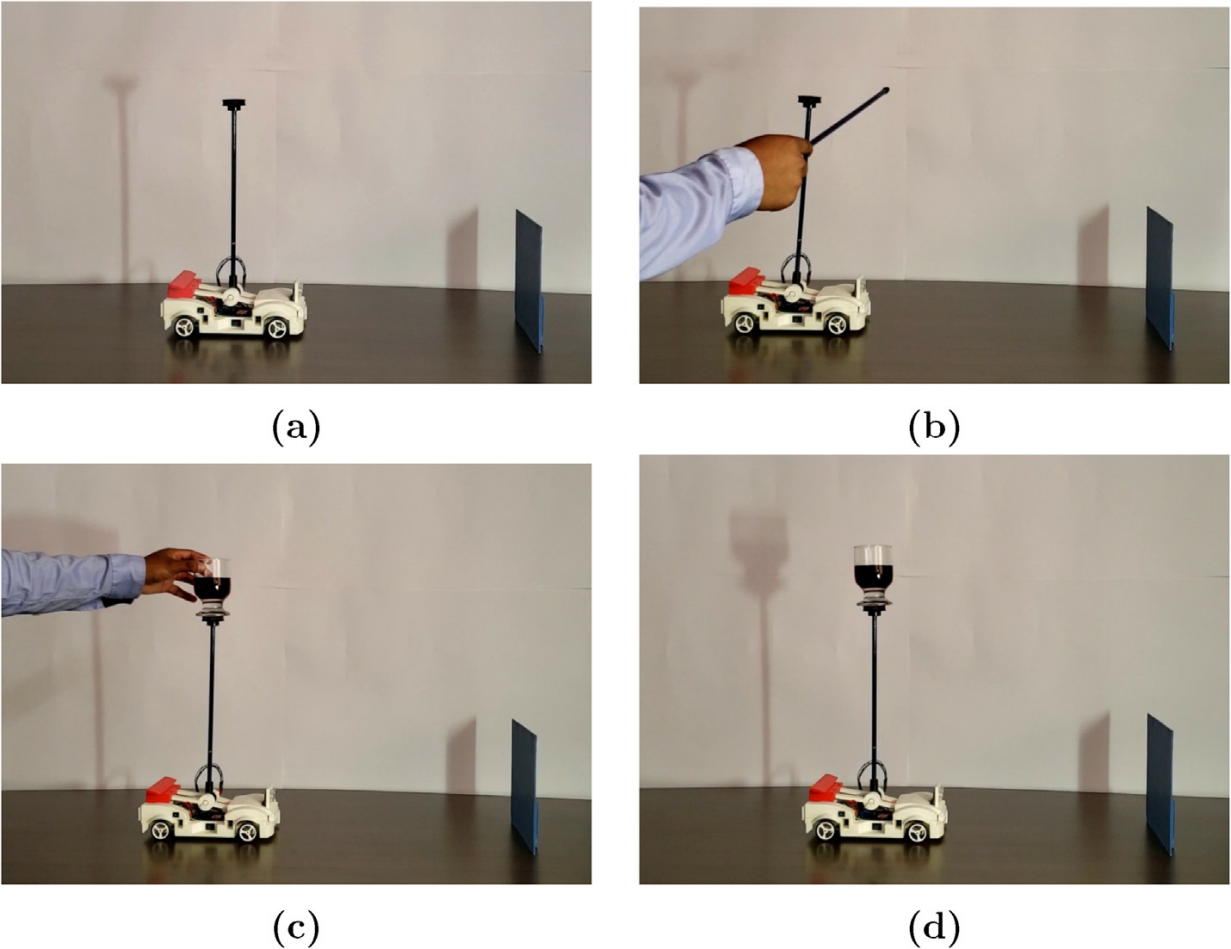
Experimental tests of MoDiCA-X: (a) test free of additional mass at the top and (b) with perturbation, (c) test with additional mass at the top and (d) keeping stability.

**Fig. 16:**
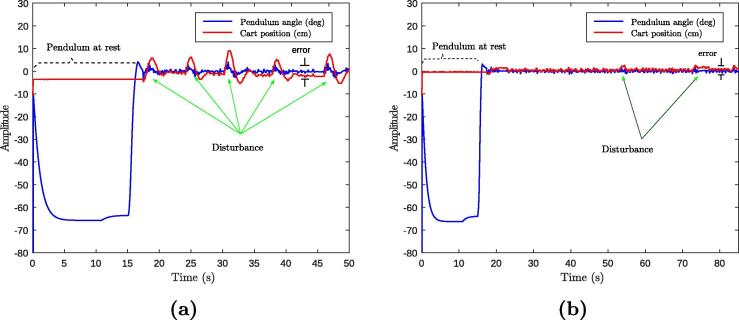
Plots of the recorded data experiments: (a) without added mass on top of the aluminum rod and (b) with additional mass (glass with liquid).

The tests were performed by placing the module on a table and in front of a vertical flat surface so that the HC-SR04 sensor could work properly; the module was initially tested free of additional mass on top Fig. 15a. The LQR (linear quadratic regulator) applied to the system showed better performance and stability (Fig. 16a), tracking the 0° angle set point and the 30 cm car position. We can see the disturbance rejection when applying an external force (Fig. 15b). A second test considered 253 grams of mass (glass with liquid) on top (Fig. 15c) and the results of the control showed to be satisfactory regarding stability in performance, tracking the desired set points and keeping the glass in vertical position and the liquid with little oscillation (Fig. 15d). Fig. 16a shows the plot of the first experiment with good disturbance rejections. Fig. 16b shows the plot of a second experiment in which the desired tracking is also guaranteed.

For this validation example, at the end of the ‘Main_code.ino’ (Section 3.2), a linear quadratic regulator (LQR) function is implemented from the line 208 and using difference equation with the methodology found in [14]. The controller gains $K_{1},K_{2},K_{3}$, and $K_{4}$, can be obtained with lqr function, available for free use in Gnu-Octave and in Scilab. Bellow, we present the LQR controller function implemented in the main microcontroller of MoDiCA-X.

**Figure:**
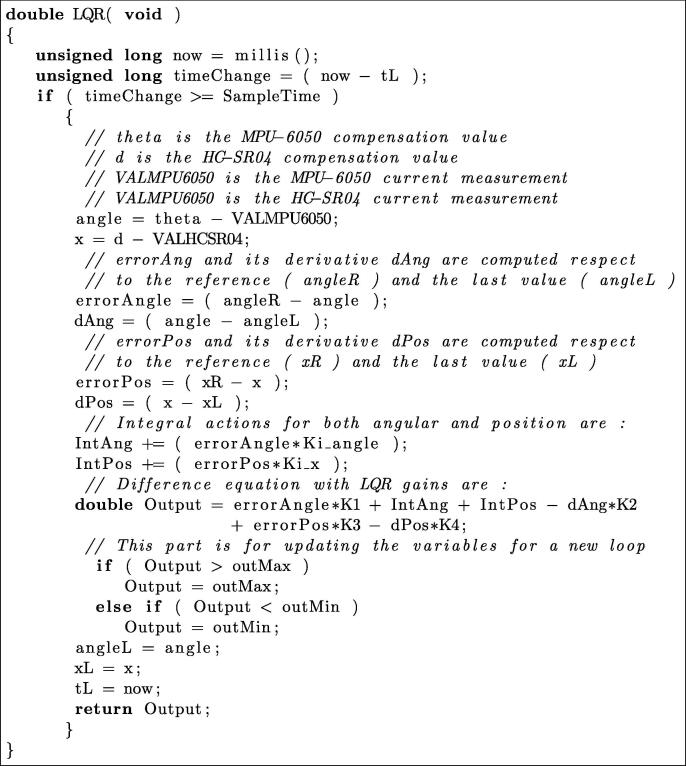

Regarding to the experiments with the LQR controller, with additional mass greater than 600 grams on top, the system tended to amplify the oscillatory phenomena, since the controller was set for a linear time invariant system. To tackle this problem of parameter variation, the control gains should be adjusted again in the algorithm or a new robust and adaptive controller should be implemented. Mass greater than 1000 grams should be avoided so as no to compromise the structural properties of the rod and bearing structure of the MoDiCA-X. In order to demonstrate the overall success of the module, videos are available via the following links:

• Experimental test on MoDiCA-X with LQR optimal control: https://youtu.be/zHyjjZ9aD2A
• Final test on MoDiCA-X with advanced control algorithm: https://www.youtube.com/watch?v=jaBRN2iAHOI
• Mounting procedures of integration: https://youtu.be/phBxw_8DVrg

In order to implement/test further advanced controllers in MoDiCA-X, the instructor or his students need only to modify or to replace the above code function with their new controller code function. Indeed, this module is to test experimentally advanced controllers previously synthesized using numerical software, such as Gnu-Octave or Scilab. The whole code shows more details in comments, such as sampling rate, register variables, calibrations functions, etc. This full code is flexible and transparent to be modified according to new requirements. All data are recorded using the auxiliary microcontroller for analysis and discussion.

## 8 Application

The main application is to help the learning process of advanced control systems in the academy and to test novel advanced control systems through the very well known car inverted pendulum system. We recommend this device due to its easy replication and construction in the academic environment using low-cost fabrication laboratory equipment, such as 3D printer, laser cutting machines and printed circuit board design tools. Further modifications, such as considering different mass, inertia and length of the rod, will be very useful experiments to be explored by researchers, instructors and academics.

Unlike the stable and minimum phase systems [15], the inverted pendulum is a more active trend topic for the control researchers [2], [9], [10], [11], [12] due to unsolved problems in related complex areas, such as in aerospace engineering, robotics, unmanned vehicles, etc.

## 9 Conclusion

MoDiCA-X is a feasible module for testing advanced control algorithms in systems characterized by under-actuated, non-minimum phase, coupling dynamics, nonlinearities, disturbances and sensor noise. We describe and share the building process of its hardware from mechanical devices using a 3D Printer to electronic printed circuit board. Moreover, the code for implementation of advanced algorithm is open source and transparent, enabling users for further modifications. The validation was performed experimentally using a linear quadratic regulator, controlling the car position and keeping the pendulum angle in the equilibrium point despite the presence of disturbances, sensor noise and model uncertainties due to mass variation on top. The total cost of the MoDiCA-X is around 100 $ US and affordable for any academic institution. Modularity, portability and flexibility turn the proposed didactic equipment attractive to be replicate.

## CRediT authorship contribution statement

**Omar Gustavo Celso Pinares Mamani:** Software, Visualization, Investigation, Validation, Writing - original draft. **Juan C. Cutipa-Luque:** Conceptualization, Methodology, Investigation, Writing - review & editing, Formal analysis, Supervision.

## Declaration of Competing Interest

The authors declare that they have no known competing financial interests or personal relationships that could have appeared to influence the work reported in this paper.
